# Longitudinal impact of the COVID-19 pandemic on the development of mental disorders in preadolescents and adolescents

**DOI:** 10.1186/s12889-023-16228-z

**Published:** 2023-07-07

**Authors:** Naomi Matsumoto, Tomoka Kadowaki, Satoe Takanaga, Yoshie Shigeyasu, Ayumi Okada, Takashi Yorifuji

**Affiliations:** 1 Department of Epidemiology, Faculty of Medicine, Dentistry and Pharmaceutical Sciences, Okayama University, 2-5-1, Shikata-Cho, Kita-Ku, Okayama-Shi, Okayama, 700-8558 Japan; 2 Department of Epidemiology, Dentistry, and Pharmaceutical Sciences, Okayama University Graduate School of Medicine, Okayama, Japan; 3 Department of Pediatrics, Dentistry and Pharmaceutical Sciences, Okayama University Graduate School of Medicine, Okayama, Japan

**Keywords:** COVID-19, Adolescence, Eating disorders, Schizophrenia, Mood disorders, Somatoform disorders, Child and adolescent mental health, Interrupted time-series

## Abstract

**Background:**

School closures and social distancing may have affected mental health among preadolescent and adolescent children, who are in a social developmental stage. Rates of anxiety, depression, and stress have been reported to have increased during the COVID-19 pandemic among teenagers worldwide. However, most studies have measured children's mental health in cross-sectional studies or short-term comparisons before and after lockdowns and school closures, and few studies have tracked the long-term effects on mental health among children and adolescents, despite the pandemic lasting more than 2 years.

**Methods:**

An interrupted time-series analysis was performed for longitudinal changes in the monthly number of new mental disorders (eating disorders, schizophrenia, mood disorders, and somatoform disorders). Using a nationwide multicenter electronic health records database in Japan, we analyzed data of patients aged 9 to 18 years from 45 facilities that provided complete data throughout the study period. The study period covered January 2017 to May 2021, defining a national school closure as an intervention event. We modeled the monthly new diagnoses of each mental disorder using a segmented Poisson regression model.

**Results:**

The number of new diagnoses throughout the study period was 362 for eating disorders, 1104 for schizophrenia, 926 for mood disorders, and 1836 for somatoform disorders. The slope of the regression line in monthly number of new diagnoses increased in the post-pandemic period for all targeted mental disorders (change in slope for eating disorders 1.05, 95% confidence interval [CI] 1.00–1.11; schizophrenia 1.04, 95% CI 1.01–1.07; mood disorders 1.04, 95% CI 1.01–1.07; and somatoform disorders 1.04 95% CI 1.02–1.07). The number of new diagnoses for schizophrenia and mood disorders increased early after school closure; while eating disorders showed an increasing trend several months later. Somatoform disorders showed a decreasing trend followed by an increasing trend. Time trends by sex and age also differed for each mental disorder.

**Conclusions:**

In the post-pandemic period, the number of new cases increased over time for eating disorders, schizophrenia, mood disorders, and somatoform disorders. The timing of increase and trends by sex and age differed for each mental disorder.

**Supplementary Information:**

The online version contains supplementary material available at 10.1186/s12889-023-16228-z.

## Introduction

The novel coronavirus infection first reported in Wuhan, China in December 2019 (COVID-19) caused a global pandemic and triggered measures in many countries to restrict the flow of people. School closures and social distancing may have affected mental health among preadolescent and adolescent children, who are in a social developmental stage.

Rates of anxiety, depression, stress, alcohol drinking, and drug use have been reported to have increased during the COVID-19 pandemic among teenagers worldwide [1, 2]. However, most studies have measured children's mental health in cross-sectional studies or short-term comparisons before and after lockdowns and school closures [3, 4], and few have examined long-term effects. One study found an increase in emergency room visits owing to acute psychiatric symptoms during lockdown and also focused on acute exacerbation of pediatric psychiatric illnesses owing to short-term stress [5]. Despite the pandemic lasting more than 2 years, few studies have tracked the long-term effects on mental health among children and adolescents. Anxiety caused by the pandemic and the continuous need for social distancing that leads to social isolation may cause persistent stress in children, which may contribute to the onset of psychiatric disorders. Analysis of long-term trends in the development of mental disorders among children and adolescents across a pandemic is vital to clarify the mechanisms of such disorders and help in prevention. However, there is a lack of published studies analyzing time trends in the development of pediatric mental disorders under pandemic conditions.

Japan is a society characterized by strong social conformity. Thus, the number of patients and deaths owing to COVID-19 has remained at a low level [6], even without lockdowns and large-scale testing that have been implemented in other countries. Such a social structure is considered a risk factor for developing mental health disorders, including mood disorders, particularly among adolescents [7]. Additionally, it has been reported that the number of patients with anorexia nervosa in Japan increased 1.6-fold in 2020 compared with 2019 [8], as did suicides among teenagers after 2020 [9].

We conducted a longitudinal analysis of the number of newly diagnosed patients with eating disorders, schizophrenia, mood disorders, and somatoform disorders among preadolescents and adolescents aged 9 to 18 years in the 38 months before and 15 months after the start of the COVID-19 pandemic using a Japanese multicenter electronic medical record (EMR) database and an interrupted time series (ITS) design.

## Methods

### Study design, participants, and setting

We conducted a natural before–after quasi-experiment to examine the number of new cases of psychiatric disorders such as eating disorders, schizophrenia, mood disorders, and somatoform disorders among preadolescents and adolescents in Japan before and after the start of the COVID-19 pandemic. Unlike insurance claims databases, EMRs are less susceptible to the influence of the working status of the targeted children's parents. Thus, given the economic downturn caused by the COVID-19 pandemic, we opted to use diagnostic information from the EMRs, which directly reflects the clinical judgment of physicians.” The data were obtained from the Real World Data (RWD) database, a nationwide Japanese EMR database operated by the Health, Clinic, and Education Information Evaluation Institute. The RWD database comprises 225 medical institutions and approximately 24.4 million EMRs covering all regions of Japan and including private and public hospitals, from large medical centers (over 1,000 beds) to clinics. The RWD database is considered highly representative of the general medical situation in Japan [10]. Furthermore, EMR diagnostic data can be obtained whether the patient is an inpatient or outpatient. In Japan, physicians enter diagnostic names as free text in EMRs, which are then converted into ICD-10 (International Classification of Diseases, Tenth Revision) codes using the ICD-10-based Standard Disease Code Master for electronic medical records (Medical Information Systems Development Center). Thus, the accuracy of ICD-10 coding in the database used in this study is reasonably assured. This database contains information on patient demographics, drug prescriptions, diagnoses, test results, and procedures. The data are collected from EMRs of each medical facility and are anonymized; each patient within the same facility is assigned a separate patient number [11].

To compare the number of preadolescent and adolescent patients with newly diagnosed mental illnesses over time, the denominator to be considered “at-risk” (the potential population to visit targeted clinics or hospitals) must be constant. Therefore, we included EMR data from facilities that provided complete data to RWD from January 2017 to May 2021 such that the number of facilities covered during the study period would remain constant. A total of 45 facilities were included, 7 with more than 500 beds, 12 with 300–500 beds, 20 with 100–300 beds, 4 with 20–100 beds, and 2 with fewer than 20 beds (including clinics). Of patients newly diagnosed with eating disorders, schizophrenia, mood disorders, and somatoform disorders at these facilities, only those between age 9 and 18 years at diagnosis were included in this longitudinal analysis of the impact of the COVID-19 pandemic on mental health among preadolescents and adolescents. For privacy reasons, only the birth year was available in the database. Therefore, to obtain each patient’s age, we calculated the age at diagnosis by assuming that all patients were born on January 1 of their birth year.

### Intervention

The Japanese Ministry of Health, Labour, and Welfare first confirmed a case of COVID-19 (a returnee from Wuhan, China) on January 16, 2020 [12]. Following that, there were only sporadic cases, mainly among returnees from Wuhan. Subsequently, the number of patients gradually increased, and social anxiety about an unknown infectious disease escalated [13]. In response to this situation and to prevent children from developing this emerging infectious disease, then Prime Minister Shinzo Abe requested that all elementary, junior high, and high schools in Japan be closed on February 27, 2020. Schools were closed in most areas after March 2 [14]. On and after mid-March, COVID-19 cases spread owing to international travel and arrivals from Europe and the United States, leading to declaration of a state of emergency in April 2020 [15, 16]. In ITS related to the COVID-19 pandemic, the declaration of a state of emergency is often used as a delimitation period for intervention [17]; however, school closure is thought to have had a more direct impact on the mental state of children [18]. Hence, we defined the period prior to February 2020 as the pre-pandemic period and that after March 2020 as the post-pandemic period. Most schools had reopened by June 1, 2020, but actions affecting the mental health of young people, such as intermittent school attendance and ongoing social distancing, continued after that date [19].

### Measurements

To examine the long-term impact of the COVID-19 pandemic on the development of psychiatric disorders in preadolescents and adolescents, the outcome of our study was set as the monthly number of newly diagnosed cases of each disorder in the pre-and post-pandemic period. We targeted several mental disorders, including eating disorders, schizophrenia, mood disorders, and somatoform disorders. Anxiety disorders, excluding somatoform disorders, were not included in this analysis because the nature of the disorder is thought to largely affect health care-seeking behavior itself [20], and it was considered challenging to assess the actual occurrence of anxiety disorders using EMR data. Monthly counts of new diagnoses for each disease and the date of diagnosis from January 2017 to May 2021 were tabulated using International Classification of Diseases Tenth Revision codes (eating disorders: F50, schizophrenia: F20, mood disorders: F30-39, and somatoform disorders: F45).

### Statistical analysis

To longitudinally assess how the COVID-19 pandemic impacted the new onset of mental health disorders in children aged 9–18 years, we conducted an ITS using the monthly number of new diagnoses for each disorder. ITS studies are considered one of the most powerful quasi-experimental designs, providing a self-controlled approach that uses both pre-and post-intervention data. This method has been widely applied in recent years because it allows observation of longitudinal pre-and post-intervention changes considering pre-intervention trends and dealing with autocorrelations, such as seasonality.

We modeled the monthly new diagnoses of each mental disorder using a segmented Poisson regression model. There were 53 time points (i.e., months) from January 2017 to May 2021, among which the 15 months from March 2020 to May 2021 were defined as the post-intervention period. A problem with ITS analysis is that outcomes in one month tend to be similar to outcomes in adjacent time points, resulting in autocorrelation and overdispersion. Therefore, we addressed autocorrelation owing to seasonality using Fourier terms and overdispersion by assuming that the variance is proportional (rather than equal) to the mean. These models were pre-designed before data acquisition.

After applying the model to the data, changes in the number of new diagnoses were visually represented by comparing the trend after intervention (nationwide school closure) to a counterfactual scenario in which the intervention would never have occurred. As an objective measure, we also estimated coefficients for the abrupt change in the monthly number of new diagnoses that occurred immediately after intervention (change in level) and the trend change over time in the monthly number of new diagnoses post-intervention compared with pre-intervention (change in slope) [21].

Lags for potential autocorrelation in the model were checked by depicting the autocorrelation function (ACF) of the residuals as the correlation between each observed and historical value at various lags and the partial ACF (PACF) as the correlation between each observed and historical value not explained by the correlation at lower order lags [22, 23]. Subgroup analyses stratified by sex and age group (preadolescence and adolescence were defined as 9–12 and 13–18 years old, respectively) were also conducted to examine differences by attributes.

All analyses were conducted using Stata version 17 (StataCorp LLC, College Station, TX, USA). This study was conducted in accordance with the Declaration of Helsinki. The Institutional Review Board at Okayama University Graduate School of Medicine, Dentistry, and Pharmaceutical Sciences approved the study protocol (No. 2107–001).

## Results

### Main results

We included 45 facilities throughout Japan that contributed data continuously without interruption during the 53-month study period. Thus, there were no missing data at the reporting facility level.

From January 2017 to May 2021, the number of new diagnoses for each disorder was as follows: 362 for eating disorders, 1104 for schizophrenia, 926 for mood disorders, and 1836 for somatoform disorders. Demographics of newly diagnosed cases are shown in Table 1, categorized according to the defined pre-and post-pandemic periods. The number of new cases in the post-pandemic period showed an increase in the proportion of girls among schizophrenia cases, those of patients diagnosed with anorexia nervosa among cases of eating disorders, and those with depressive episodes among mood disorders cases.

**Table 1:** Baseline characteristics of newly diagnosed patients with mental health disorders, pre- and post-COVID-19

	Patients, No. (%)		
	Whole time series	Pre-pandemic	Post-pandemic
**Eating disorders**			
Total	362 (100)	240 (100)	122 (100)
Sex			
Male	73 (20.2)	49 (20.4)	24 (19.7)
Female	289 (79.8)	191 (79.6)	98 (80.3)
Age			
9–12 years	126 (34.8)	85 (35.4)	41 (33.6)
13–18 years	236 (65.2)	155 (64.6)	81 (66.4)
ICD-10			
Anorexia nervosa (F50.0)	101 (27.9)	61 (25.4)	40 (32.8)
Eating disorder, unspecified (F50.9)	222 (61.3)	149 (62.1)	73 (59.8)
Other (F50.1, F50.2, F50.3, F50.4, F50.0, F50.8)	39 (10.8)	30 (12.5)	9 (7.4)
**Schizophrenia**			
Total	1104 (100)	765 (100)	339 (100)
Sex			
Male	463 (41.9)	348 (45.5)	115 (33.9)
Female	641 (58.1)	417 (54.5)	224 (66.1)
Age			
9–12 years	392 (35.5)	281 (36.7)	111 (32.7)
13–18 years	712 (64.5)	484 (63.3)	228 (67.3)
**Mood disorders**			
Total	926 (100)	586 (100)	340 (100)
Sex			
Male	364 (39.3)	241 (41.1)	123 (36.2)
Female	562 (60.7)	345 (58.9)	217 (63.8)
Age			
9–12 years	170 (18.4)	104 (17.8)	66 (19.4)
13–18 years	756 (81.6)	482 (82.2)	274 (80.6)
ICD-10			
Manic episode (F30)	33 (3.6)	21 (3.6)	12 (3.5)
Bipolar disorder (F31)	71 (7.7)	52 (8.9)	19 (5.6)
Depressive episode (F32)	666 (70.9)	407 (69.5)	249 (73.2)
Persistent mood disorders (F34)	149 (16.1)	97 (16.6)	52 (15.3)
Other (F33, F38, F39)	17 (1.9)	9 (1.6)	8 (2.4)
**Somatoform disorders**			
Total	1836 (100)	1347 (100)	489 (100)
Sex			
Male	722 (39.3)	515 (38.2)	207 (42.3)
Female	1114 (60.7)	832 (61.8)	282 (57.7)
Age			
9–12 years	567 (30.9)	415 (30.8)	152 (31.1)
13–18 years	1269 (69.1)	932 (69.2)	337 (68.9)

*ICD-10* International Classification of Diseases, Tenth Revision

**Fig. 1:**
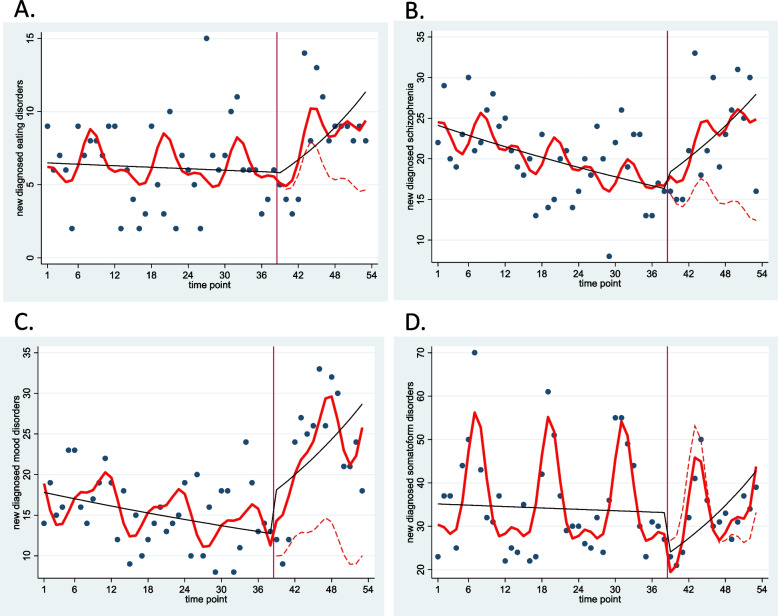
Monthly reports of newly diagnosed cases of mental disorders (dots) with trend lines. **A** Eating disorders. **B** Schizophrenia. **C** Mood disorders. **D** Somatoform disorders. Solid red line: predicted trend based on the seasonally adjusted regression model. Black line: de-seasonalized trend. Dashed red line: counterfactual trend assuming no intervention

A segmented Poisson regression analysis was then applied to evaluate the monthly number of new diagnoses for each disease during the study period (Table 2 and Fig. 1). In the case of eating disorders, the number of new diagnoses per month trended upward for a few months following school closure and remained at a high level (change in slope 1.05, 95% confidence interval [CI] 0.998–1.11). For schizophrenia and mood disorders, an increase in the monthly number of new diagnoses was observed relatively soon after school closure (change in level for schizophrenia 1.14, 95% CI 0.86–1.51 and mood disorders 1.43, 95% CI 1.05–1.95) and continued to increase after that (change in slope for schizophrenia 1.04, 95% CI 1.01–1.07 and mood disorders 1.04, 95% CI 1.01–1.07). In the case of somatoform disorders, the monthly number of new diagnoses temporarily decreased with the closing of schools (change in level 0.73, 95% CI 0.59–0.90) and then increased (change in slope 1.04, 95% CI 1.02–1.07).

**Table 2:** Changes in number of newly diagnosed mental health disorders in pre- and post-pandemic periods

	mean [SD]		Estimated coefficient [95% CI]		
	Pre-COVID	Post-COVID	Baseline incidence rate	Level change ^a^	Slope change ^b^
Eating disorders	240 [7.61]	122 [9.28]	6.60 [4.92–8.85]	1.00 [0.59–1.69]	1.05 [1.00–1.11]
Schizophrenia	765 [21.26]	339 [24.22]	24.48 [21.14–28.35]	1.14 [0.86–1.51]	1.04 [1.01–1.07]
Mood disorders	586 [16.53]	340 [24.84]	18.21 [15.32–21.64]	1.43 [1.05–1.95]	1.04 [1.01–1.07]
Somatoform disorders	1347 [39.39]	489 [34.22]	35.70 [31.97–39.86]	0.73 [0.59–0.90]	1.04 [1.02–1.07]

*SD* Standard deviation; *CI* Confidence interval

^a^Level change refers to an abrupt level change in pre-COVID-19 versus post-COVID-19 periods

^b^Slope change refers to slope change over time in pre-COVID-19 versus post-COVID-19 periods

The residual ACFs and PACFs at most lags in each model did not exceed the 95% CI, which indicated that the autocorrelation was negligible and did not violate the models, for both models (eFigure 1).

### Subgroup analyses by sex and age

**Fig. 2:**
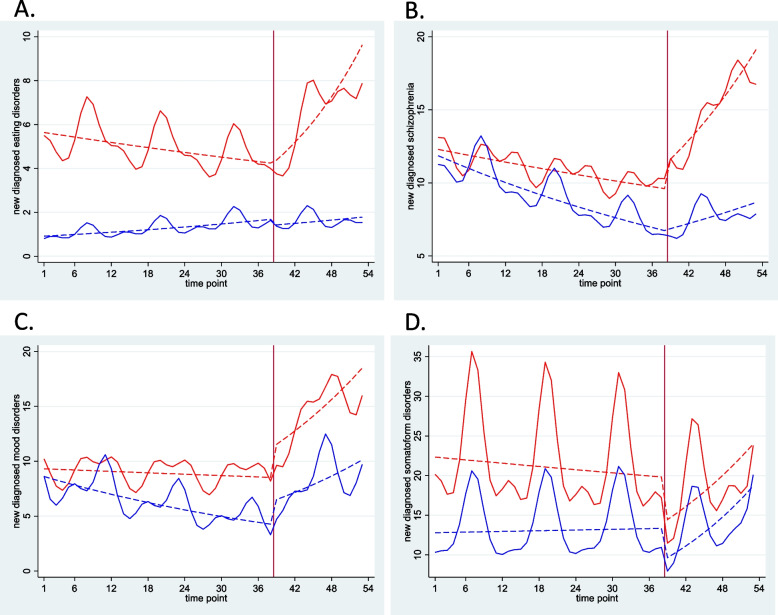
Monthly reports of newly diagnosed mental disorders with dashed trend lines, stratified by sex. **A** Eating disorders. **B** Schizophrenia. **C** Mood disorders. **D** Somatoform disorders. Red line: female, blue line: male

A greater increase in change in the slope of the regression line for the monthly number of new diagnoses of eating disorders and schizophrenia in the post-pandemic period was observed in girls (change in slope for eating disorders 1.07, 95% CI 1.01–1.13 and schizophrenia 1.04, 95% CI 1.01–1.08). The monthly number of new diagnoses of mood disorders and somatoform disorders showed a similar trend over time for both sexes (Fig. 2, eTable 1).

**Fig. 3:**
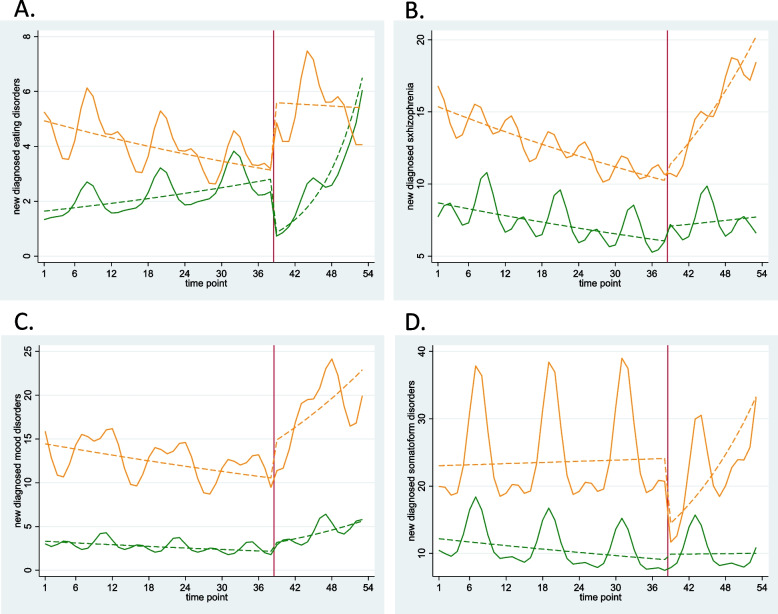
Monthly reports of newly diagnosed cases of mental disorders (dots) with trend lines, stratified by age group. **A** Eating disorders. **B** Schizophrenia. **C** Mood disorders. **D** Somatoform disorders. Yellow line: preadolescents, green line: adolescents

The findings of subgroup analyses stratified by age are shown in Fig. 3 and eTable 2. The slope of the regression line in the monthly number of new diagnoses of schizophrenia, mood disorders, and somatoform disorders showed a more significant increase during the post-pandemic period in the adolescent group (13–18 years old) (change in slope for schizophrenia 1.05, 95% CI 1.02–1.09; mood disorders 1.04, 95% CI 1.00–1.08, and somatoform disorders 1.06, 95% CI 1.03–1.09). The monthly number of new diagnoses of eating disorders increased immediately after school closure in the adolescent group (change in level 1.80, 95% CI 1.02–3.09) and remained at high levels; this number decreased temporarily in the preadolescent group (9–12 years old) (change in level 0.30, 95% CI 0.12–0.79) and then increased over time (change in slope 1.14, 95% CI 1.04–1.25).

## Discussion

We compared time trends in the monthly number of preadolescents and adolescents aged 9–18 years in Japan newly diagnosed with eating disorders, schizophrenia, mood disorders, and somatoform disorders in the pre-and post-pandemic period using an ITS analysis with a Japanese multicenter EMR database. Although the slope of the regression line in the monthly number of new diagnoses increased in the post-pandemic period for all targeted mental disorders, schizophrenia and mood disorders showed an increase in the number of new diagnoses relatively early after school closure whereas eating disorders showed an increasing trend several months later. Somatoform disorders showed a decreasing trend followed by an increasing trend. These trends were relatively more pronounced in girls with eating disorders and schizophrenia. There were no apparent differences in the trends of new diagnoses by sex for mood disorders and somatoform disorders. The post-pandemic linear increase over time in monthly number of new diagnoses of schizophrenia, mood disorders, and somatoform disorders was more pronounced in adolescents than in preadolescents.

Adolescents, in transition to adulthood, are a vulnerable population [24], and their mental health has been strongly affected by pandemic-related anxiety as well as school closures, the need for social distancing, and increased time spent accessing social networking sites [25]. The impact of the COVID-19 pandemic on the mental health of preadolescents and adolescents has been widely documented, suggesting increased anxiety and depression [26, 25] Larger impacts have been reported in girls and adolescents [2, 27]. However, most past studies were either cross-sectional or compared periods before and after lockdown. Few studies have examined long-term, chronological effects of the pandemic, even though social restrictions and behavioral changes caused by the pandemic have continued for more than 2 years. Previous studies on new cases of the mental disorders investigated in our study have also reported increases in the incidence of eating disorders and mood disorders after the start of the pandemic; however, few of these studies have examined long-term effects. [2, 28, 29] There are even fewer studies regarding the pandemic’s impact on the number of new cases of schizophrenia and somatoform disorders in teenagers [30, 31]. Our results support previous findings, suggesting a post-pandemic increase in the number of new cases of the investigated mental disorders. Our findings also provide further insight into the long-term, chronological impact of the pandemic using an ITS design.

There are some limitations in this study. Although teenagers with a history of psychiatric illness may be more susceptible to the impact of the pandemic on mental health [32], we did not account for past comorbidities of other psychiatric illnesses; therefore, the differences in risk between individuals with and without a history of psychiatric illness could not be assessed. There are reports of increased psychiatric disorders owing to COVID-19 [33], but we did not consider a history of COVID-19 infection. However, the number of teenagers with COVID-19 in Japan remained below 0.05% of the total population of teenagers until May 2021 [34, 35], the period covered by this study. Thus, its influence is considered negligible. The subgroup analyses by age group may be affected by the assumption that everyone is born on January 1, owing to missing information for the exact date of birth. Additionally, measurement bias may exist, such as discrepancies in the diagnosis name among EMR data and the actual clinical condition. Owing to the potential impact of changes in parental employment status resulting from the economic downturn during the COVID-19 pandemic, there is a possibility that the insurance coverage of the children under study may have been affected. Therefore, we used EMR data from healthcare institutions instead of an insurance claims database. However, it should be noted that whereas the RWD used in this study have only been validated in the three domains of cardiovascular diseases, hemophilia, and cancer [11, 36, 37] there has been no specific validation for the field of psychiatry. Consequently, concerns regarding the validity of the information used in this study remain. Moreover, in patients who change medical facility, their data may be recorded elsewhere as data for a different patient. However, because we fixed the denominator (i.e., the medical institution providing the data), such non-differential misclassification should not strongly distort the results in pre- and post-comparisons of the overall diagnoses. The most important barrier to conducting ITS is the presence of time-varying confounders, such as refraining from medical visits. In fact, in terms of the actual number of new diagnoses, a temporary decrease was observed for all mental health disorders covered in this study immediately after school closure. A previous study from the same database, an interrupted time series analysis of the number of new monthly diagnoses of atopic dermatitis, found a 20% decrease, probably due to refraining from seeing a doctor, after the simultaneous closure of all schools nationwide, keeping a similar lower level throughout the observation period [38]. However, all mental health disorders targeted in this study showed an increasing trend over time, suggesting an increase in each disease incidence itself, even if underestimated. Data on the severity of each mental health disorder were not considered in this study. We used data from Japan, therefore, our findings are not generalizable to other countries.

## Conclusion

We analyzed the longitudinal impact of the COVID-19 pandemic on the incidence of mental health disorders in preadolescents and adolescents in this ITS study using a multicenter EMR database in Japan. The number of new cases increased over time after the start of the pandemic for eating disorders, schizophrenia, mood disorders, and somatoform disorders. The timing of the increase and trends by sex and age differed for each mental health disorder. Our findings suggest that mental health support is needed for preadolescents and adolescents who are mentally vulnerable populations during a pandemic, especially older adolescents and women.

### Supplementary Information

**Additional file 1.**

## Data Availability

The RWD data that support the findings of this study are available from the Real World Data Co., Ltd. but restrictions apply to the availability of these data, which were used under license for the current study, and so are not publicly available. The Data are however available to approved researchers for a fee; for more information, visit: https://rwdata.co.jp or contact the corresponding author.

## Abbreviations

ACF: Autocorrelation function
CI: Confidence interval
COVID-19: Coronavirus disease 2019
EMR: Electronic medical records
ITS: Interrupted time series
PACF: Partial autocorrelation function
RWD: Real World Data

## References

[1] Jones EAK, Mitra AK, Bhuiyan AR (2021). Impact of COVID-19 on Mental Health in Adolescents: A Systematic Review. Int J Environ Res Public Health.

[2] Samji H, Wu J, Ladak A, Vossen C, Stewart E, Dove N (2022). Review: Mental health impacts of the COVID-19 pandemic on children and youth – a systematic review. Child Adolesc Ment Health.

[3] Cost KT, Crosbie J, Anagnostou E, Birken CS, Charach A, Monga S (2022). Mostly worse, occasionally better: impact of COVID-19 pandemic on the mental health of Canadian children and adolescents. Eur Child Adolesc Psychiatry.

[4] Viner R, Russell S, Saulle R, Croker H, Stansfield C, Packer J (2022). School Closures During Social Lockdown and Mental Health, Health Behaviors, and Well-being Among Children and Adolescents During the First COVID-19 Wave: A Systematic Review. JAMA Pediatr.

[5] Shankar LG, Habich M, Rosenman M, Arzu J, Lales G, Hoffmann JA (2022). Mental Health Emergency Department Visits by Children Before and During the COVID-19 Pandemic. Acad Pediatr.

[6] Situation report on COVID-19 (in Japanese). Ministry of Health, Labour and Welfare, Japan. https://www.mhlw.go.jp/stf/covid-19/kokunainohasseijoukyou_00006.html. Accessed 12 Jul 2022.

[7] Lebedina-Manzoni M, Lotar M, Ricijas N. (2011) Peer Pressure in Adolescence: boundaries and possibilities. Univ Zagreb.

[8] National Center for Child Health and Development. Survey of Children’s Minds in the Corona Disaster (in Japanese). 2021. https://www.ncchd.go.jp/press/2021/211021.html. Accessed 12 Jul 2022.

[9] Ministry of Health, Labour and Welfare Statistics on Suicides: Annual Situation (in Japanese). 2022. https://www.mhlw.go.jp/stf/seisakunitsuite/bunya/hukushi_kaigo/seikatsuhogo/jisatsu/jisatsu_year.html. Accessed 6 Jun 2023.

[10] Real World Data, Co., Ltd. https://rwdata.co.jp/. Accessed 8 Jun 2022.

[11] Ono Y, Taneda Y, Takeshima T, Iwasaki K, Yasui A (2020). Validity of Claims Diagnosis Codes for Cardiovascular Diseases in Diabetes Patients in Japanese Administrative Database. Clin Epidemiol.

[12] Novel Coronavirus – Japan. https://www.who.int/emergencies/disease-outbreak-news/item/2020-DON236. Accessed 27 Apr 2022.

[13] Open data (in Japanese). Ministry of Health, Labour and Welfare, Japan. https://www.mhlw.go.jp/stf/covid-19/open-data.html. Accessed 29 Jun 2022.

[14] Yamamura E, Tsustsui Y (2021). School closures and mental health during the COVID-19 pandemic in Japan. J Popul Econ.

[15] Al HSM, Saulam J, Kanda K, Ngatu NR, Hirao T (2021). Trends in COVID-19 Outbreak in Tokyo and Osaka from January 25 to May 6 2020 a Joinpoint Regression Analysis of the Outbreak Data. Jpn J Infect Dis.

[16] Press Conference. https://www.mhlw.go.jp/stf/seisakunitsuite/bunya/newpage_00032.html. Accessed 27 Apr 2022.

[17] Yoshioka E, Hanley SJB, Sato Y, Saijo Y (2022). Impact of the COVID-19 pandemic on suicide rates in Japan through December 2021: An interrupted time series analysis. Lancet Reg Heal - West Pacific.

[18] Hawrilenko M, Kroshus E, Tandon P, Christakis D (2021). The Association Between School Closures and Child Mental Health During COVID-19. JAMA Netw Open.

[19] Marchi J, Johansson N, Sarkadi A, Warner G (2021). The Impact of the COVID-19 Pandemic and Societal Infection Control Measures on Children and Adolescents’ Mental Health: A Scoping Review. Front Psychiatry.

[20] Ganson KT, Weiser SD, Tsai AC, Nagata JM (2020). Associations between Anxiety and Depression Symptoms and Medical Care Avoidance during COVID-19. J Gen Intern Med.

[21] Kontopantelis E, Doran T, Springate DA, Buchan I, Reeves D. Regression based quasi-experimental approach when randomisation is not an option: interrupted time series analysis. BMJ. 2015;350. 10.1136/bmj.h2750 PMC4460815 26058820

[22] Bernal JL, Cummins S, Gasparrini A (2017). Interrupted time series regression for the evaluation of public health interventions: a tutorial. Int J Epidemiol.

[23] Schaffer AL, Dobbins TA, Pearson SA (2021). Interrupted time series analysis using autoregressive integrated moving average (ARIMA) models: a guide for evaluating large-scale health interventions. BMC Med Res Methodol.

[24] Larsen B, Luna B (2018). Adolescence as a neurobiological critical period for the development of higher-order cognition. Neurosci Biobehav Rev.

[25] Guessoum SB, Lachal J, Radjack R, Carretier E, Minassian S, Benoit L (2020). Adolescent psychiatric disorders during the COVID-19 pandemic and lockdown. Psychiatry Res.

[26] Nearchou F, Hennessy E, Flinn C, Niland R, Subramaniam SS (2020). Exploring the Impact of COVID-19 on Mental Health Outcomes in Children and Adolescents: A Systematic Review. Int J Environ Res Public Health.

[27] Chen F, Zheng D, Liu J, Gong Y, Guan Z, Lou D (2020). Depression and anxiety among adolescents during COVID-19: A cross-sectional study. Brain Behav Immun.

[28] Agostino H, Burstein B, Moubayed D, Taddeo D, Grady R, Vyver E (2021). Trends in the Incidence of New-Onset Anorexia Nervosa and Atypical Anorexia Nervosa Among Youth During the COVID-19 Pandemic in Canada. JAMA Netw Open.

[29] Haripersad YV, Kannegiesser-Bailey M, Morton K, Skeldon S, Shipton N, Edwards K (2021). Outbreak of anorexia nervosa admissions during the COVID-19 pandemic. Arch Dis Child.

[30] O’Donoghue B, Collett H, Boyd S, Zhou Y, Castagnini E, Brown E (2022). The incidence and admission rate for first-episode psychosis in young people before and during the COVID-19 pandemic in Melbourne. Australia Aust N Z J Psychiatry.

[31] Turco R. Pediatric emergency care admissions for somatic symptom disorders during the COVID 19 pandemic. 2022. 10.21203/RS.3.RS-1665503/V1. 10.1007/s00431-022-04687-2 PMC9716529 36459226

[32] Fountoulakis KN, Karakatsoulis G, Abraham S, Adorjan K, Ahmed HU, Alarcón RD (2022). Results of the COVID-19 mental health international for the general population (COMET-G) study. Eur Neuropsychopharmacol.

[33] Xie Y, Xu E, Al-Aly Z. Risks of mental health outcomes in people with covid-19: cohort study. BMJ. 2022;376. 10.1136/bmj-2021-068993 PMC8847881 35172971

[34] Learning from Data - New Coronavirus Infectious Disease Information - (in Japanese). Ministry of Health, Labour and Welfare Japan. https://covid19.mhlw.go.jp/. Accessed 6 Jul 2022.

[35] Statistics Bureau Website/Population Estimates (as of October 1, 2021) (in Japanese). https://www.stat.go.jp/data/jinsui/2021np/index.html. Accessed 6 Jul 2022.

[36] Fujiwara T, Miyakoshi C, Kanemitsu T, Okumura Y, Tokumasu H (2021). Identification and Validation of Hemophilia-Related Outcomes on Japanese Electronic Medical Record Database (Hemophilia-REAL V Study). J Blood Med.

[37] Fujiwara T, Kanemitsu T, Tajima K, Yuri A, Iwasaku M, Okumura Y (2022). Accuracy of algorithms to identify patients with a diagnosis of major cancers and cancer-related adverse events in an administrative database: a validation study in an acute care hospital in Japan. BMJ Open.

[38] Matsumoto N, Kadowaki T, Takanaga S, Ikeda M, Yorifuji T (2022). Impact of COVID-19 pandemic-associated reduction in respiratory viral infections on childhood asthma onset in Japan. J Allergy Clin Immunol Pract.

