# Correction: longitudinal impact of the COVID-19 pandemic on the development of mental disorders in preadolescents and adolescents

**DOI:** 10.1186/s12889-023-17499-2

**Published:** 2024-01-02

**Authors:** Naomi Matsumoto, Tomoka Kadowaki, Satoe Takanaga, Yoshie Shigeyasu, Ayumi Okada, Takashi Yorifuji

**Affiliations:** 1 Department of Epidemiology, Faculty of Medicine, Dentistry and Pharmaceutical Sciences, Okayama University, 2-5-1, Shikata-Cho, Kita-Ku, 700-8558 Okayama-Shi, Okayama, Japan; 2 Department of Epidemiology, Dentistry, and Pharmaceutical Sciences, Okayama University Graduate School of Medicine, Okayama, Japan; 3 Department of Pediatrics, Dentistry and Pharmaceutical Sciences, Okayama University Graduate School of Medicine, Okayama, Japan

**Correction to: BMC Public Health (2023) 23:1308**

10.1186/s12889-023-16228-z

The original publication of this article contained an incorrect funding section. The incorrect and correct information is listed in this correction article. The original article has been updated.

**Incorrect**

This study was supported by The Japan Foundation for Pediatric Research Grant No. 20–017 and JSPS KAKENHI Grant No. JP20K23195. Data were also provided by the Health, Clinic, and Education Information Evaluation Institute HCEI; Kyoto, Japan), a not-for-profit research service foundation, in cooperation with the Real World Data Co., Ltd. (Kyoto, Japan). The sponsors had no role in the design and conduct of the study; collection, management, analysis, and interpretation of the data; preparation, review, and approval of the manuscript; or in the decision to submit the manuscript for publication.

Japan Society for the Promotion of Science,JP20K23195,The Japan Foundation for Pediatric Research,20 − 017.

**Correct**

This study was supported by The Japan Foundation for Pediatric Research Grant No. 20–017 and JSPS KAKENHI Grant No. JP20K23195. Data were also provided by the Health, Clinic, and Education Information Evaluation Institute HCEI; Kyoto, Japan), a not-for-profit research service foundation, in cooperation with the Real World Data Co., Ltd. (Kyoto, Japan). The sponsors had no role in the design and conduct of the study; collection, management, analysis, and interpretation of the data; preparation, review, and approval of the manuscript; or in the decision to submit the manuscript for publication.

This work was supported by The Japan Foundation for Pediatric Research Grant No. 20 − 017 and JSPS KAKENHI Grant No. JP20K23195 and JP23K16329.

