# Supplementary material for: Longitudinal impact of the COVID-19 pandemic on the development of mental disorders in preadolescents and adolescents
Source: BMC Public Health. 2023 Jul 7;23:1308. doi: 10.1186/s12889-023-16228-z (PMC10327305; doi:10.1186/s12889-023-16228-z)
Supplement: Supplementary file 1 — Additional file 1. [file 12889_2023_16228_MOESM1_ESM.docx]

| **eTable 1. Changes in number of newly diagnosed mental health disorders, pre- and post-COVID-19 (by sex)** | | | | | | |
| --- | --- | --- | --- | --- | --- | --- |
|  |  | **mean [SD]** | | **Estimated coefficient [95% CI]** | | |
|  |  | **Pre-COVID** | **Post-COVID** | **Baseline incidence rate** | **Level change ^a^** | **Slope change ^b^** |
| **Eating disorders** | |  |  |  |  |  |
|  | **Male** | 49 [1.29] | 24 [1.60] | 0.91 [0.15-1.83] | 0.84 [0.28-2.49] | 1.00 [0.89-1.12] |
|  | **Female** | 191 [5.03] | 98 [6.53] | 5.76 [4.28-7.77] | 1.04 [0.60-1.82] | 1.07 [1.01-1.13] |
| **Schizophrenia** | |  |  |  |  |  |
|  | **Male** | 348 [9.16] | 115 [7.67] | 12.14 [9.95-14.82] | 1.04 [0.68-1.58] | 1.03 [0.99-1.08] |
|  | **Female** | 417 [10.97] | 224 [14.93] | 12.40 [10.13-15.17] | 1.22 [0.85-1.75] | 1.04 [1.01-1.08] |
| **Mood disorders** | |  |  |  |  |  |
|  | **Male** | 241 [6.34] | 123 [8.20] | 8.93 [7.02-11.35] | 1.56 [0.97-2.49] | 1.05 [1.00-1.10] |
|  | **Female** | 345 [9.08] | 217 [14.47] | 9.44 [7.49-11.88] | 1.36 [0.92-1.99] | 1.04 [1.00-1.08] |
| **Somatoform disorders** | |  |  |  |  |  |
|  | **Male** | 515 [13.55] | 207 [13.80] | 12.96 [10.82-15.53] | 0.72 [0.52-1.01] | 1.05 [1.01-1.08] |
|  | **Female** | 832 [21.89] | 282 [18.80] | 22.74 [19.78-26.13] | 0.73 [0.55-0.96] | 1.04 [1.01-1.07] |
| SD, standard deviation; CI, confidence interval. | | |  |  |  |  |
| ^a^ Level change refers to an abrupt level change in pre-COVID-19 versus post-COVID periods | | | | |  |  |
| ^b^ Slope change refers to slope change over time in pre-COVID-19 versus post-COVID-19 periods. | | | | |  |  |

| **eTable 2. Changes in number of newly diagnosed mental health disorders, pre- and post-COVID-19 (by age)** | | | | | | |
| --- | --- | --- | --- | --- | --- | --- |
|  |  | **mean [SD]** | | **Estimated coefficient [95% CI]** | | |
|  |  | **Pre-COVID** | **Post-COVID** | **Baseline incidence rate** | **Level change ^a^** | **Slope change ^b^** |
| **Eating disorders** | |  |  |  |  |  |
|  | **Ages 9–12 years** | 85 [2.24] | 41 [2.73] | 1.64 [0.98-2.75] | 0.30 [0.12-0.79] | 1.14 [1.04-1.25] |
|  | **Ages 13–18 years** | 155 [5.23] | 81 [5.40] | 5.16 [3.70-6.92] | 1.80 [1.02-3.09] | 1.01 [0.95-1.07] |
| **Schizophrenia** | |  |  |  |  |  |
|  | **Ages 9–12 years** | 281 [7.39] | 111 [7.40] | 8.84 [6.89-11.34] | 1.18 [0.73-1.91] | 1.02 [0.97-1.07] |
|  | **Ages 13–18 years** | 484 [12.74] | 228 [15.20] | 15.58 [13.09-18.54] | 1.12 [0.80-1.57] | 1.05 [1.02-1.09] |
| **Mood disorders** | |  |  |  |  |  |
|  | **Ages 9–12 years** | 104 [2.74] | 66 [4.40] | 3.37 [2.25-5.04] | 1.49 [0.74-3.01] | 1.05 [0.99-1.12] |
|  | **Ages 13–18 years** | 482 [12.68] | 274 [18.27] | 14.78 [12.18-17.94] | 1.43 [1.01-2.02] | 1.04 [1.00-1.08] |
| **Somatoform disorders** | |  |  |  |  |  |
|  | **Ages 9–12 years** | 415 [10.92] | 152 [10.13] | 12.45 [10.38-14.93] | 1.10 [0.78-1.55] | 1.01 [0.97-1.05] |
|  | **Ages 13–18 years** | 932 [24.53] | 337 [22.47] | 23.34 [20.29-26.86] | 0.60 [0.46-0.79] | 1.06 [1.03-1.09] |
| SD, standard deviation; CI, confidence interval. | | | |  |  |  |
| ^a^ Level change refers to an abrupt level change in pre-COVID-19 versus post-COVID-19 periods. | | | | |  |  |
| ^b^ Slope change refers to slope change over time in pre-COVID-19 versus post-COVID-19 periods. | | | | |  |  |


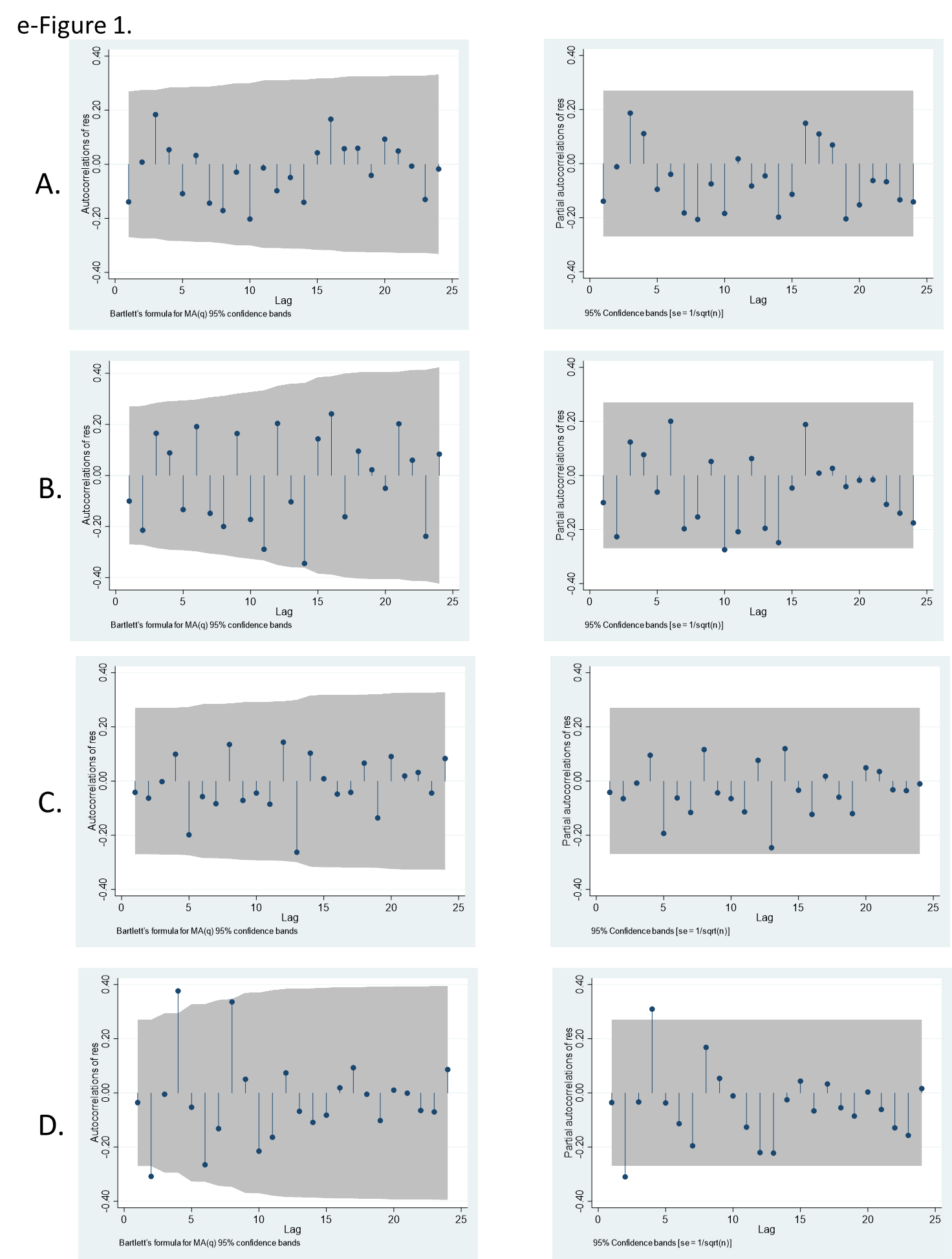
eFigure 1. Visual plots of the autocorrelation function (ACF, left) and partial autocorrelation function (PACF, right). A) Eating disorder model. B) Schizophrenia model. C) Mood disorder model. D) Somatoform disorder model.
